# From modesty to modernity: The story of plastic surgery in Qatar

**DOI:** 10.5339/qmj.2025.67

**Published:** 2025-09-22

**Authors:** Khalifa Al Alawi, Mohamed Badie Ahmed, Alreem Al-Khayarin, Abeer Alsherawi, Habib Al Basti

**Affiliations:** 1 Plastic Surgery Department, Hamad General Hospital, Hamad Medical Corporation, Doha, Qatar; 2 Plastic & Reconstructive Surgery department, Khoula Hospital, Muscat, Oman; 3 College of Medicine, QU Health, Qatar University, Doha, Qatar *Email: khalifakmk@gmail.com

**Keywords:** Qatar, plastic surgery, health care system, evolution

## Abstract

Qatar, a small yet wealthy state on the Arabian Peninsula, has witnessed significant changes in its healthcare system over the years. This article examines the development of healthcare in Qatar, with a focus on plastic surgery services, starting from traditional medicine to becoming one of the most advanced systems in the region. Key milestones include the establishment of Hamad Medical Corporation and the Ministry of Public Health, which have played important roles in organizing and improving healthcare services, in line with Qatar National Vision 2030.

The article also highlights the growth of plastic surgery services, which began in the 1970s with limited resources and visiting specialists. Over time, these services have expanded with the recruitment of skilled surgeons and the introduction of advanced facilities. The integration of burn care, the growth of reconstructive and aesthetic procedures, and the launch of a plastic surgery residency program demonstrate Qatar’s efforts to enhance this field.

The private sector has also contributed to this progress, offering a range of services that complement the public healthcare system. These developments reflect Qatar’s commitment to providing quality healthcare and supporting the well-being of its people. This article traces the key steps in the history of healthcare and plastic surgery in Qatar, showing how they have evolved to meet the needs of the population.

## 1. INTRODUCTION

Qatar is a small yet exceptionally wealthy state situated on the Arabian Peninsula. The name “Qatar” is believed to originate from the Arabic word Qatrah, meaning “a drop,” a reference to the country’s connection to seawater and its historical role in maritime trade. Additionally, the Greek geographer Ptolemy, in the second century CE, produced one of the earliest maps depicting the peninsula, referring to it as “Catara.” This term persisted in historical texts and evolved over time to become the modern name “Qatar.”^1,2^

The history of Qatar dates back to ancient times, with evidence of early human habitation in the Gulf region.^3^ Over the centuries, the land has experienced a rich and diverse history, coming under the rule of various empires and states. However, it is widely agreed that the modern history of Qatar begins in the 18th century. In 1868, Sheikh Mohammed bin Thani, the founder of the Al Thani royal family, signed a treaty with Great Britain, recognizing Qatar as a state under the protection of the British Empire. In 1971, Sheikh Khalifa bin Hamad Al Thani declared Qatar an independent state, marking the beginning of a new era of modernization.^4^

## 2. THE DAWN OF A HEALTH CARE SYSTEM

The evolution of Qatar’s healthcare system represents a remarkable transformation, transitioning from traditional medical practices to one of the most advanced healthcare systems in the region. Historically, healthcare in Qatar was dominated by traditional medicine, with treatments such as cauterization and herbal remedies being commonplace. During this period, barbers often performed minor medical procedures, while herbalists provided natural treatments to address various ailments.^5^

**Figure 1.**
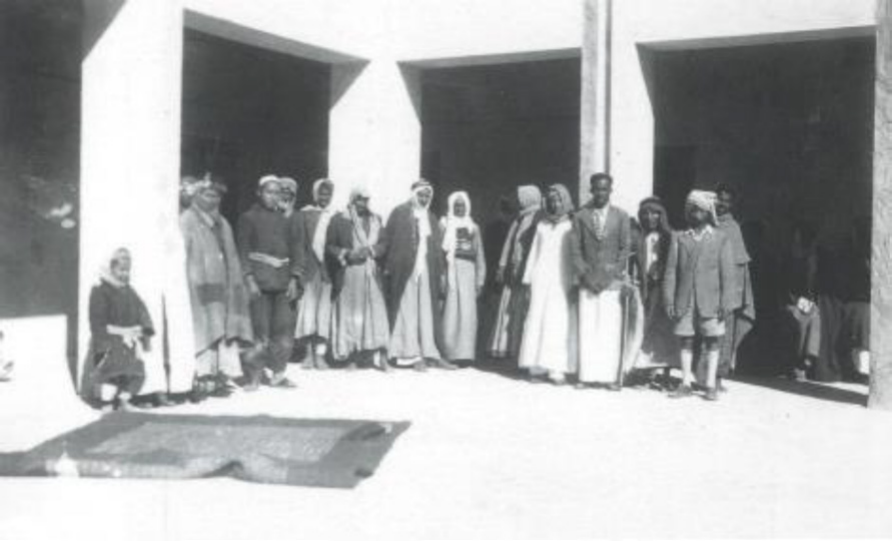
Group of citizens standing in front of Qatar’s First Hospital, 1940s.^6^

The landscape began to shift in 1943 when Sheikh Abdullah bin Jassim Al-Thani initiated Qatar’s first hospital project (Figure 1).^6^ This facility, which opened in 1947, featured a modest 12-bed capacity and was staffed by rotating American physicians. The discovery of oil in the 1940s catalyzed significant socioeconomic transformations, including advancements in healthcare infrastructure, driven by newfound economic prosperity.^5,7^

**Figure 2.**
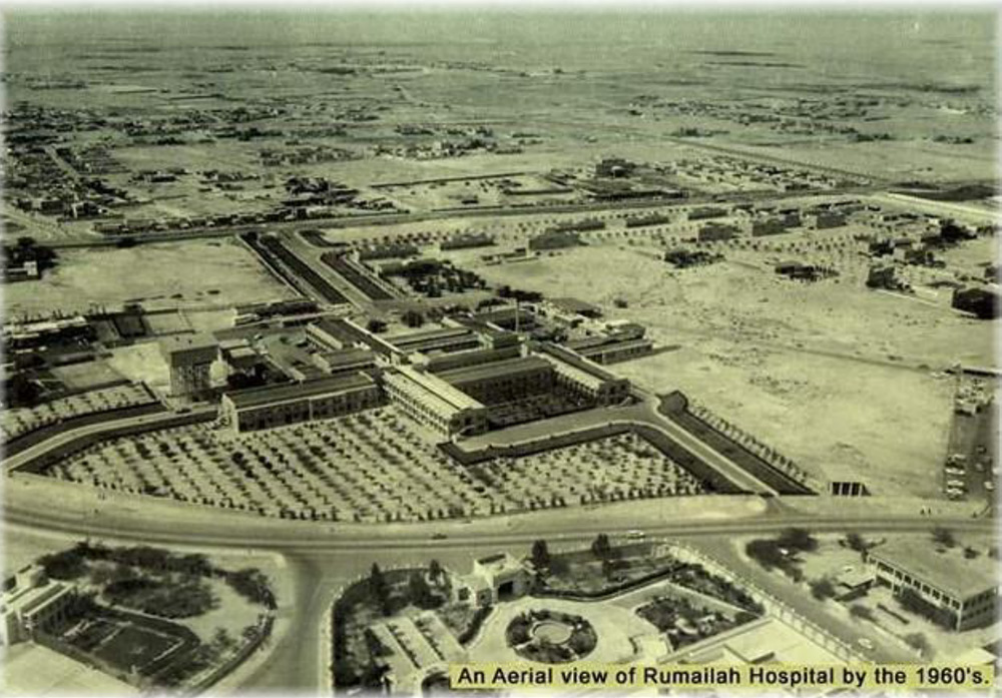
Aerial view of Al Rumailah Hospital in Doha, Qatar, in the 1960s.

The establishment of Al Rumailah Hospital (RH) in 1957 was a pivotal moment in Qatar’s healthcare journey, as it marked the opening of the country’s first government hospital (Figure 2).^6,8^ This milestone laid the foundation for a more structured approach to public health in Qatar. In 1979, the healthcare system took another transformative step with the founding of Hamad Medical Corporation (HMC) through an Emiri Decree. HMC became a cornerstone of Qatar’s healthcare evolution, unifying services under one umbrella to enhance care coordination and maximize resource efficiency. Over the years, HMC has grown into a robust institution, managing multiple specialized hospitals and serving as the backbone of Qatar’s public healthcare system.^9^ Another significant leap forward came in 1993 with the establishment of the Ministry of Public Health, also initiated by an Emiri Decree. This development was a testament to Qatar’s dedication to creating a comprehensive, organized, and future-focused healthcare system. The Ministry was tasked with shaping the healthcare landscape by organizing the sector and defining its key competencies, further solidifying Qatar’s commitment to the well-being of its population.^10^

## 3. MEDICAL EDUCATION, THE CORNERSTONE OF ANY HEALTH SYSTEM

Qatar’s leadership understands the importance of medical education and has invested significantly in developing local expertise. In 2001, Weill Cornell Medicine – Qatar became the country’s first medical school. It offers a full medical curriculum that leads to an MD degree from Weill Cornell University, making history as the first American university to award its MD degree outside the United States.^11^ In 2014, Qatar University College of Medicine was established to support local medical education further and meet the growing need for doctors.^12^ This addition strengthened Qatar’s healthcare system by increasing opportunities for medical training within the country. Sidra Medicine, which opened in 2018, is another example of Qatar’s focus on specialized healthcare. This modern 400-bed facility combines patient care, medical education, and research. It addresses complex medical issues, particularly for women and children, and plays a vital role in advancing healthcare in Qatar.^13^ The Ministry of Public Health continues to ensure that Qatar’s healthcare system remains world-class. Recent efforts include investments in telemedicine and digital health services, which keep the system up to date with global trends.^9^

From its humble beginnings, Qatar’s healthcare system has grown into a leader in the region. Investments in education, infrastructure, and specialized services reflect the country’s strong commitment to improving healthcare for all its citizens.^14^ These efforts align with Qatar National Vision 2030, which aims to create a sustainable, advanced society by 2030. The vision focuses on human, social, economic, and environmental development to ensure a high quality of life for everyone in Qatar.^15^

## 4. PLASTIC SURGERY SERVICES

The development of plastic surgery services in Qatar mirrors the broader growth of the country’s healthcare system. These services began modestly in the early 1970s at RH, where burn care became the first specialized area within the field of plastic surgery. At the time, treatment was limited to basic wound dressings and minimal surgical interventions. Qatar had no resident plastic surgeons, relying instead on visiting specialists. Notably, two Irish plastic surgeons provided crucial care by visiting RH for 2 weeks every 3 months. Their work included cleft lip and palate surgeries and reconstructive procedures, addressing significant gaps in patient care. In 1980, Professor Abdulfatah Abdullah, an Egyptian burn and plastic surgeon, was recruited as Qatar’s first permanent plastic surgeon at RH. However, the services remained in their infancy and operated as part of the general surgery department.

The turning point came in 1984 when Dr. Saad El-Ekiaby, another Egyptian plastic surgeon, was recruited. That same year, Qatar established its first dedicated plastic surgery department at the newly built Hamad General Hospital (HGH), marking a significant milestone. The department began offering a broader range of services, including burn surgery, reconstructive surgery, and aesthetic surgery.^16^

The evolution of plastic surgery in Qatar gained momentum with the return of the first two trained Qatari plastic surgeons: Dr. Habib Al Basti (FRCS) in 1995 and Dr. Talal Al Hetmi (Facharzt [The German Board] of Plastic and Reconstructive Surgery). Their expertise and leadership transformed the department. In 1996, Dr. Habib introduced hand surgery to the department, a field previously managed by orthopedic surgeons [Figures 7–9 (Supplementary Material)]. He also pioneered microsurgery in the same year, equipping the department with the necessary tools and infrastructure.

**Figure 3.**
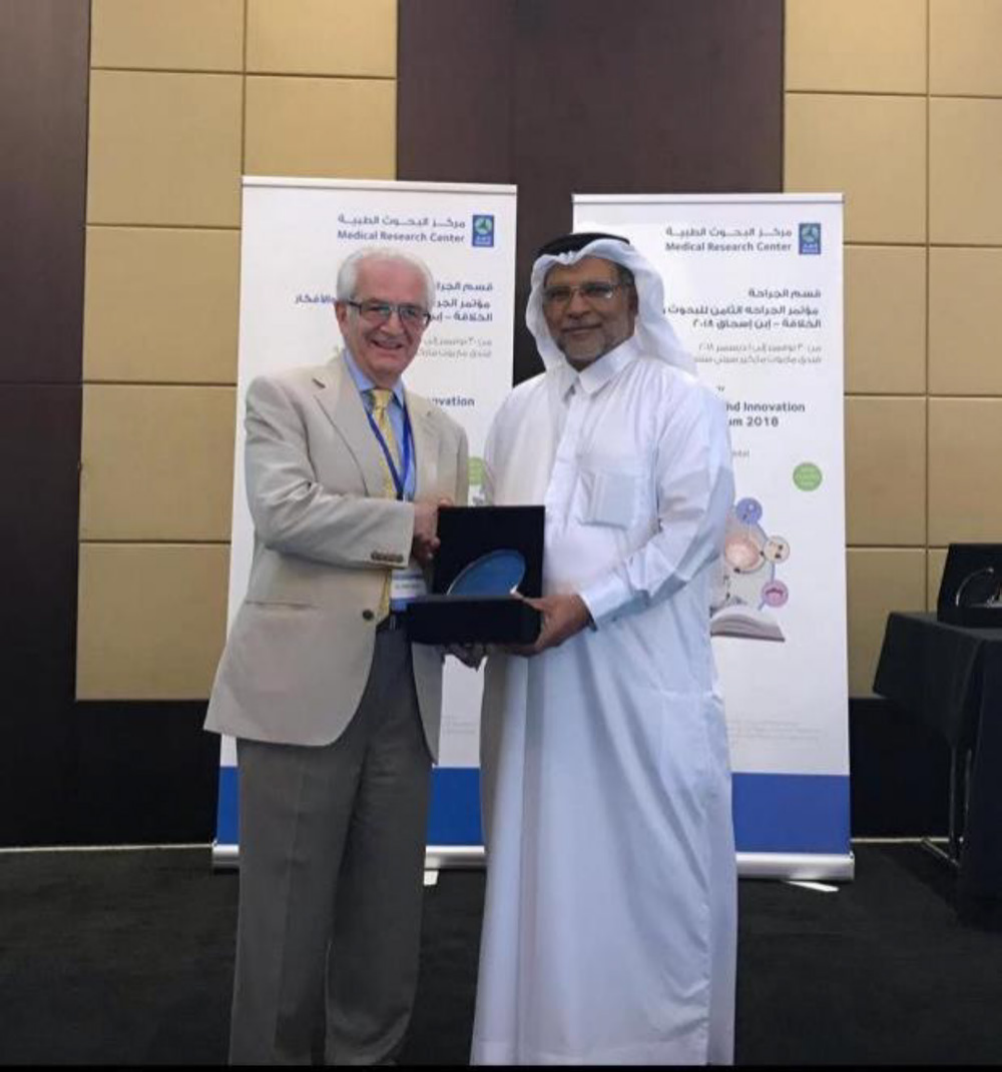
Dr Habib Al Basti (right) with Professor Foad Nahai (left) during his visit to HMC in 2018.

In 1999, the Plastic Surgery Department relocated from HGH back to RH, where it underwent further growth. Over the next two decades, the services expanded across multiple HMC hospitals. This expansion included the establishment of a dedicated Burn Unit at Al Wakra Hospital and the opening of additional units at other facilities. To strengthen local expertise, the department leaders invited world-renowned plastic surgeons as visiting consultants. Esteemed specialists such as Foad Nahai, Allen Gilbert, Al Aly, Mutaz Habal, JP Hong, Aivar Bracka, Michel Chammas, Sven Olof Wikstrom, Patrik Velander, Magnus Becker, and Victoria Rose contributed significantly to the advancement of plastic surgery in Qatar [Figure 3; Figures 10 and 11 (Supplementary Material)].

In 2019, the main plastic surgery department moved from RH to HGH and the Surgical Specialty Center. This relocation marked the latest chapter in the remarkable growth of plastic surgery services in Qatar, which continue to thrive and serve the needs of the population.

The department participates in advancing the knowledge and skills in the rejoin and hosts several regional and international conferences, including the Conference of the Pan Arab Association of Plastic, Reconstructive, Aesthetic, and Burn Surgery (2012) and Conference of G.C.C. Association of Plastic, Reconstructive, Aesthetic, and Burn Surgery (2012).

## 5. FROM SEAWATER TO A STATE-OF-THE-ART UNIT, THE DEVELOPMENT OF BURN SERVICES

**Figure 4.**
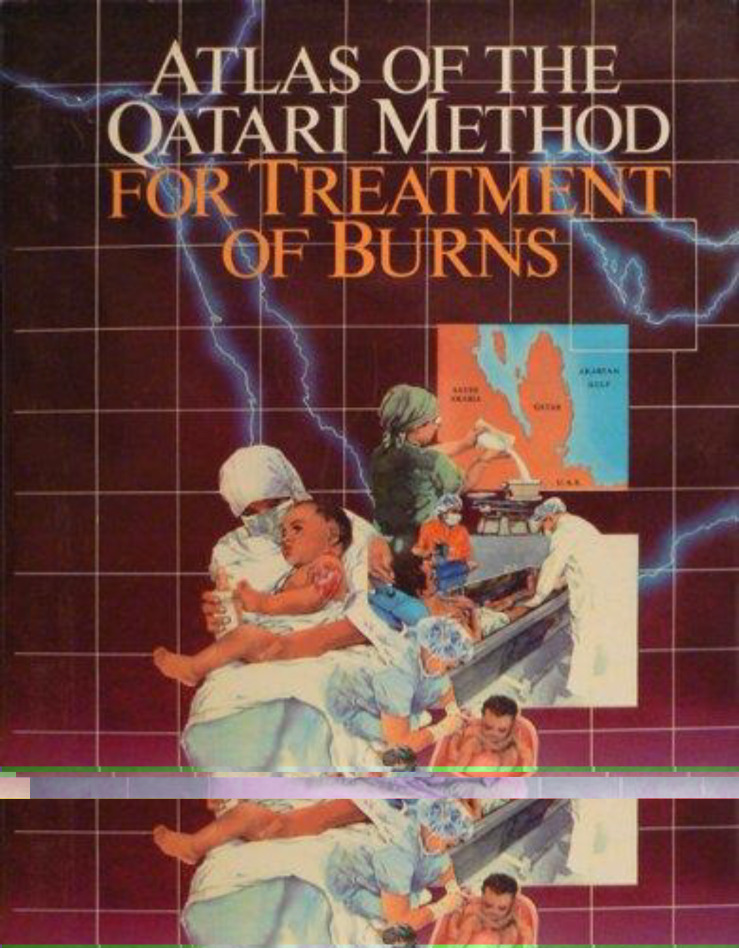
*Atlas of the Qatari Method for Treatment of Burns,* published on January 1, 1986.^18^

The early treatment of burns was inspired by traditional Qatari wound care practices for children post-circumcision, where the child would be bathed in seawater for several days after the procedure. This concept was later adopted and refined by the burn team at RH, who developed a specific protocol for burn care. The key figures in the burn team were Dr. Abdulla Al-Baker, Qatar’s first burn surgeon, who pioneered burn care in the country, and Dr. Aref Al-Ghoul, who played a crucial role in developing and advancing burn services, significantly improving patient care. Drs Al-Baker, Al-Ghoul, and their colleagues formalized this approach into a comprehensive protocol, which they published in a book titled *Atlas of the Qatari Method for Treatment of Burns*. Subsequently, they published a review article documenting the outcomes of 1,500 burn cases managed using the Qatari method (Figure 4).^17,18^

The Qatari approach to burn care involved keeping burn wounds exposed in a controlled environment with the room temperature regulated at 29 °C to support recovery and prevent heat loss. Patients underwent bathing in a diluted saline solution (prepared by dissolving 4–5 g of sodium chloride per liter of tap water) twice daily for 20 to 30 minutes, except for specific areas like the hands, feet, buttocks, and perineum, which were cleaned 3 to 4 times a day, and the face, which was washed 4 to 6 times daily. To manage pain, patients with extensive burns received intramuscular Phenergan before bathing. During baths, dead tissue and exudates were carefully removed, and continuous saline dressings were applied between bathing sessions for deep burns. Physiotherapy, including both active and passive joint and muscle exercises, was provided under the guidance of nurses and physiotherapists to maintain mobility. Wound swabs were taken for culture and sensitivity testing on the second, third, and fourth days and then at 3- to 4-day intervals. Systemic antibiotics were prescribed when clinically necessary, while topical antibiotics were avoided. Hemoglobin and serum protein levels were regularly assessed and corrected using blood transfusions and protein supplementation. Skin grafting was performed only when required, typically around the third week of treatment.^18^

The burn team was integrated into the plastic surgery department at RH from the 1980s until 2014. In 2014, the newly constructed Al Wakrah Hospital was equipped with a fully developed burn unit designed to handle all burn referrals from across Qatar. The unit includes regular burn beds, as well as specialized Pediatric Intensive Care Unit (PICU) and Surgical Intensive Care Unit (SICU) beds for burn patients. It incorporates advanced burn surgical techniques and utilizes a wide variety of modern dressing materials to ensure optimal patient care.^16^

## 6. CURRENT STATUS

**Figure 5.**
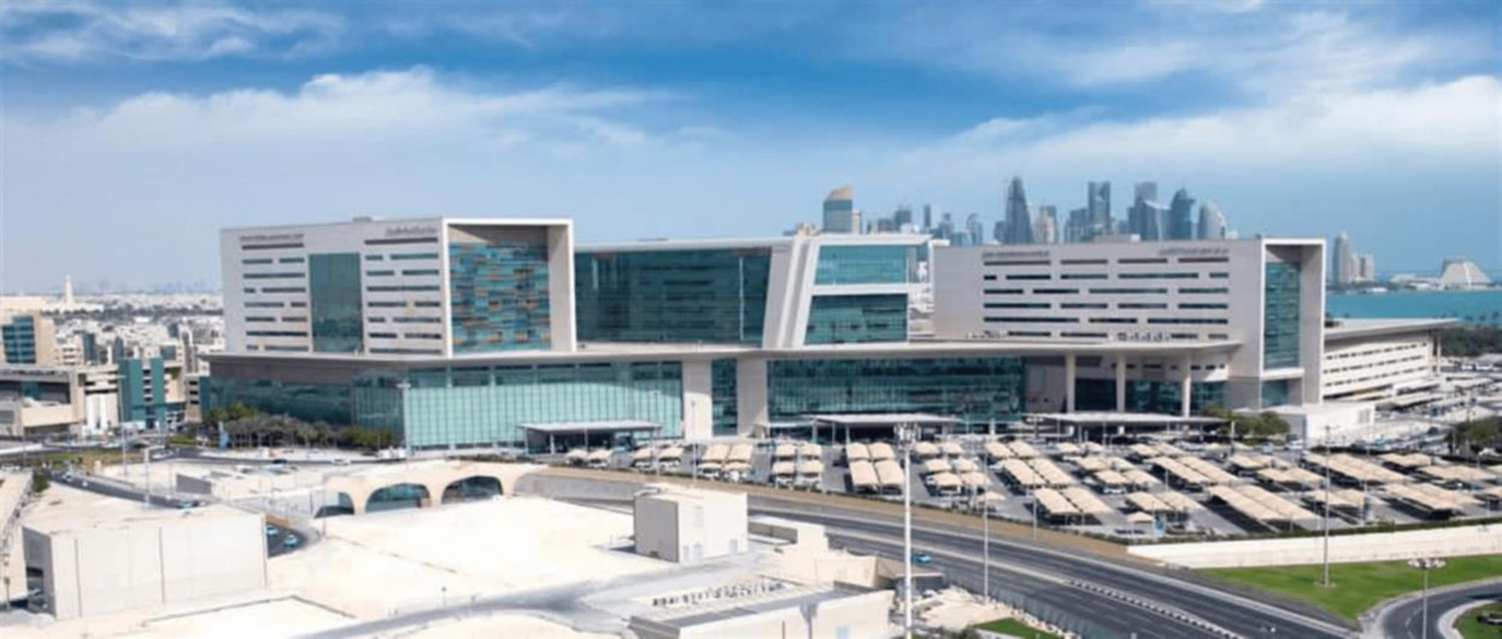
Ambulatory Care Center within Hamad Medical City in Doha.^19^

Currently, plastic surgery services are provided across multiple HMC facilities, including HGH, Al Wakra Hospital, Al Khor Hospital, Cuban Hospital, and Hazem Mebaireek Hospital. Additionally, the department offers its services at the Ambulatory Care Center (ACC) for the daycare major and minor procedures (Figure 5).^19^ Over the past decades, the scope of practice has expanded to include more specialized care, such as advanced hand and microsurgery, breast reconstruction, melanoma surgery, head and neck reconstruction, and lower limb reconstruction, among others. Sidra Medicine, which operates independently of HMC, specializes in advanced pediatric and craniofacial plastic surgery [Figures 12 and 13 (Supplementary Material)]. It is worth noting that Qatar’s public health system is unique in offering the full range of aesthetic procedures performed by consultants free of charge to all Qatari citizens. Furthermore, the department envisions further expanding its services to additional institutions and creating specialized units to enhance patient care. The Qatar Society of Plastic, Burn, and Reconstructive Surgeons is currently chaired by Dr. Habib Al Basti, with Dr. Talal Al Hetmi serving as the deputy [Figure 14 (Supplementary Material)]. The society operates under the umbrella of the Qatar Doctors Association. Qatar is also a partner in the International Society of Aesthetic Plastic Surgery (ISAPS), actively participating in advancing the field of aesthetic and reconstructive surgery.

## 7. PRIVATE SECTOR

In 1997/1998, Dr. Habib Al Basti established Qatar’s first private plastic surgery practice. Since then, the field of plastic surgery in Qatar’s private healthcare sector has experienced remarkable growth, reflecting the expansion of public services and the increasing demand for both cosmetic and reconstructive procedures. Today, Qatar is home to numerous private hospitals and specialized clinics offering a comprehensive range of services. Prominent private hospitals, such as The View Hospital, Al-Ahli Hospital, and Al Emadi Hospital, among others, exemplify this growth. This parallel development of private plastic surgery services complements the public sector, providing patients with a broader spectrum of options for specialized care.

## 8. PLASTIC SURGERY RESIDENCY

Before the establishment of the plastic surgery residency program, the department comprised consultants, specialists, and service residents, who were assigned to one or more teams to assist in delivering patient care. Between 2010 and 2011, the Plastic Surgery Department in Qatar, represented by Dr Talal Al Hetmi and Dr Mahmoud Al Thalathini, played a pivotal role in creating the plastic surgery program under the Arab Board of Health Specializations. This program officially launched on January 1, 2012, with an inaugural cohort of five residents.^20^

Dr Habib and Dr Talal served as examiners in 2012 and 2013. Since then, Dr. Habib has continued to represent Qatar and serve as an examiner to the present day.

The plastic surgery residency program is a multi-institutional, integrated, 6-year training program. During this period, residents undergo comprehensive training in both para-plastic and plastic surgery. Clinical rotations were initially conducted at RH, where residents gained hands-on experience. In addition to clinical training, the program offers opportunities to participate in both basic and clinical science research at the HMC Research Center.

The fully integrated program is structured into blocks designed to provide a strong foundation in surgical patient management. Residents progressively take on greater responsibilities according to their training level while gaining specialized knowledge and skills in surgical subspecialties closely related to plastic surgery. The curriculum ensures residents gain experience across all areas of the discipline, including general surgery, orthopedic surgery, pediatric surgery, critical care medicine, burns management, dermatology, maxillofacial surgery, ophthalmology, otorhinolaryngology, and accident and emergency medicine.

The program accepts an average of two to three residents per year, welcoming both national and international applicants. Since its inception, it has been graduating an average of two to three residents annually. Some graduates have continued their careers in the department, while others have secured placements in international fellowship programs across the United States, the United Kingdom, Europe, and other countries worldwide. The first cohort to graduate from the program comprised one resident in 2017.

**Figure 6.**
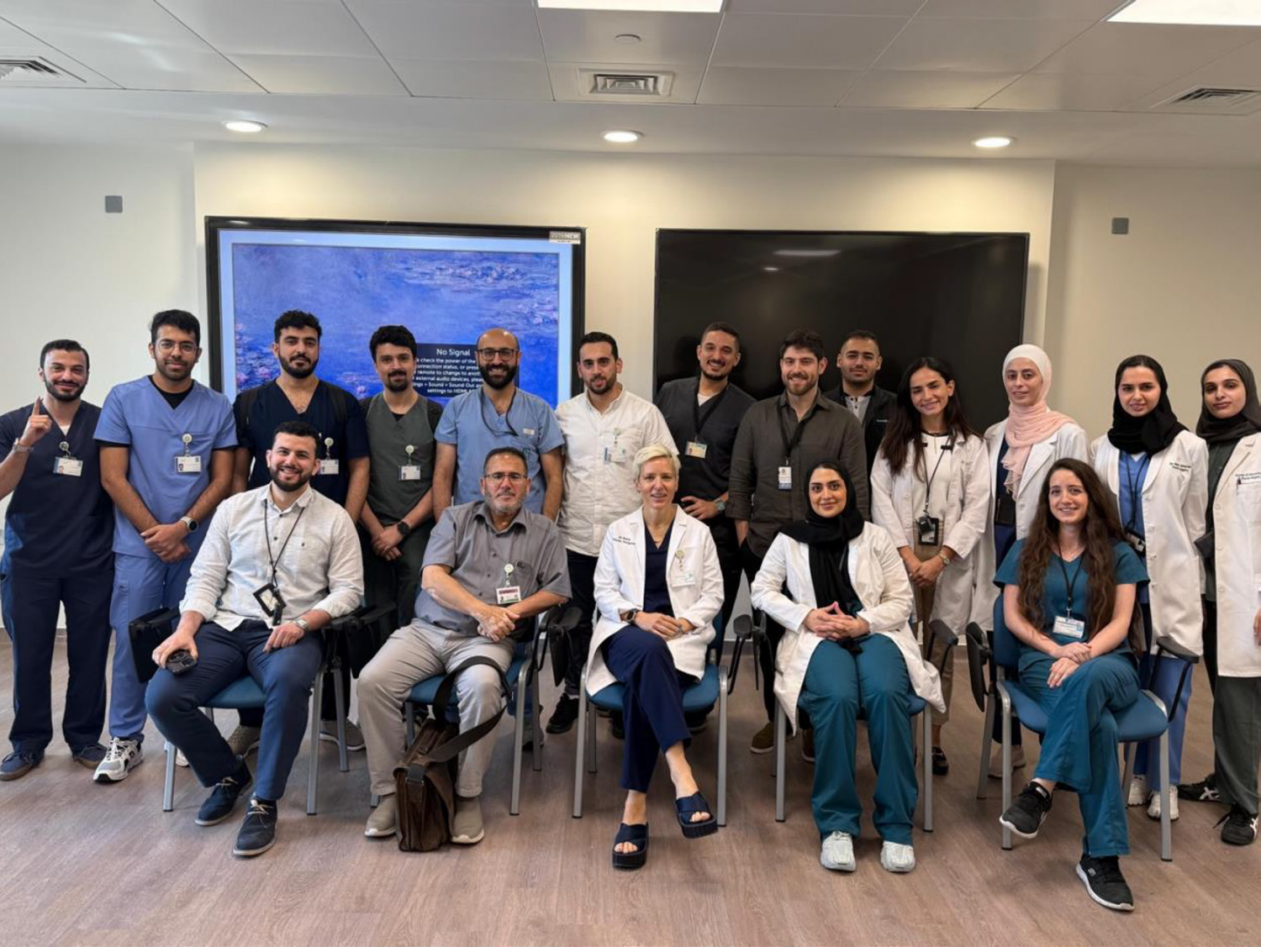
Dr. Victoria Rose (front row, middle seat), a visiting breast plastic surgeon, with a group of plastic residents on October 31, 2024.

Over the years, the program continued to grow under the leadership of the first fully trained Qatari female plastic surgeons, Dr. Sara Al Harami [Arab Board Certification (plast.)], the program director, and Dr. Abeer Alsherawi (Facharzt of Plastic and Reconstructive Surgery/Facharzt of Hand Surgery), the associate program director. The program provides high-quality training and includes workshops such as cadaveric flap dissection workshops, as well as collaborations with national universities to offer research courses [Figure 6; Figures 15 – 18 (Supplementary Material)]. In 2021, the program reached yet another milestone by receiving Accreditation Council for Graduate Medical Education-International (ACGME-I) accreditation. In 2025, the program was enrolled into the Qatari Board of Medical Specialties (QBMS) under the leadership of Dr Abeer Alsherawi.^21^

## 9. CONCLUSION

The evolution of plastic surgery in Qatar reflects the nation’s broader commitment to advancing its healthcare system. From its modest beginnings in the 1970s with visiting specialists to the establishment of a dedicated plastic surgery department and a residency program, Qatar has made remarkable strides in this field. The integration of burn care, the introduction of microsurgery, and the expansion of both public and private sector services have positioned Qatar as a leader in plastic and reconstructive surgery in the region. These advancements align with Qatar National Vision 2030, ensuring continued progress in medical education, innovation, and patient care.

## ACKNOWLEDGMENT

We sincerely thank Dr. Aref Al Ghoul, Dr. Talal Al Hetmi, Dr. Mahmoud Al Thalathini, Dr. Ahmed Taha, Dr. Jimmy Thomas, Dr Noora Al Tamimi, Dr. Mohamed Muneer El-Noor, Dr. Mutaz Abuelgasim, and Dr. Mahmoud El Sharkawy for their invaluable contributions. We appreciate their time, insights, and support, without which this work would not have been possible.
